# Progress in liquid crystal chemistry

**DOI:** 10.3762/bjoc.5.48

**Published:** 2009-10-07

**Authors:** Sabine Laschat

**Affiliations:** 1 Institut für Organische Chemie, Universität Stuttgart, Pfaffenwaldring 55, D-70569 Stuttgart, Germany

Since their discovery in 1888 by the botanist Friedrich Reinitzer, who studied the melting behavior of cholesteryl benzoates, liquid crystals have developed from a mere curiosity to a highly interdisciplinary research field, with LC displays being the most important application to date [1–2]. However, besides the use of thermotropic liquid crystals for display technology many other applications will probably be on the market in the near future, such as organic light emitting diodes, photovoltaic devices, organic field effect transistors, gas sensors etc. [3–8]. One of the major issues in liquid crystal research today is still the poor knowledge of structure-property relationships and thus the synthesis of whole series of structurally related compounds is required in order to allow the design of liquid crystalline and other physical properties. It is the goal of this thematic series “Progress in Liquid Crystal Chemistry” to give the reader of *Beilstein Journal of Organic Chemistry* a flavor of the synthetic challenges of liquid crystal research, as well as interdisciplinary aspects from material science, physics, physical and theoretical chemistry. It is a great pleasure to serve as a guest editor for this thematic series, where leading experts from all over the world have contributed original research papers and accounts that deal with diverse topics such as phthalocyanine-fullerene hybrid materials for photovoltaic devices, functional metallomesogens, coaxial electrospinning of liquid crystal-containing microfibers, banana-discotic hybrid systems, ionic liquid crystals, relations between molecular length distribution and formation of smectic phases, nematic phase engineering by using V-shaped molecules, saddle-shaped columnar systems displaying anomalous odd-even effects, theoretical studies on the origin of chirality transfer in liquid crystalline host-guest systems, liquid crystalline carboranes and dyes and discotic phenanthrene quinones. Many new exciting discoveries in this diverse research area are to be expected in the near future and our aim with this Thematic Series is to attract a new audience to this topic.

Sabine Laschat

Guest Editor
